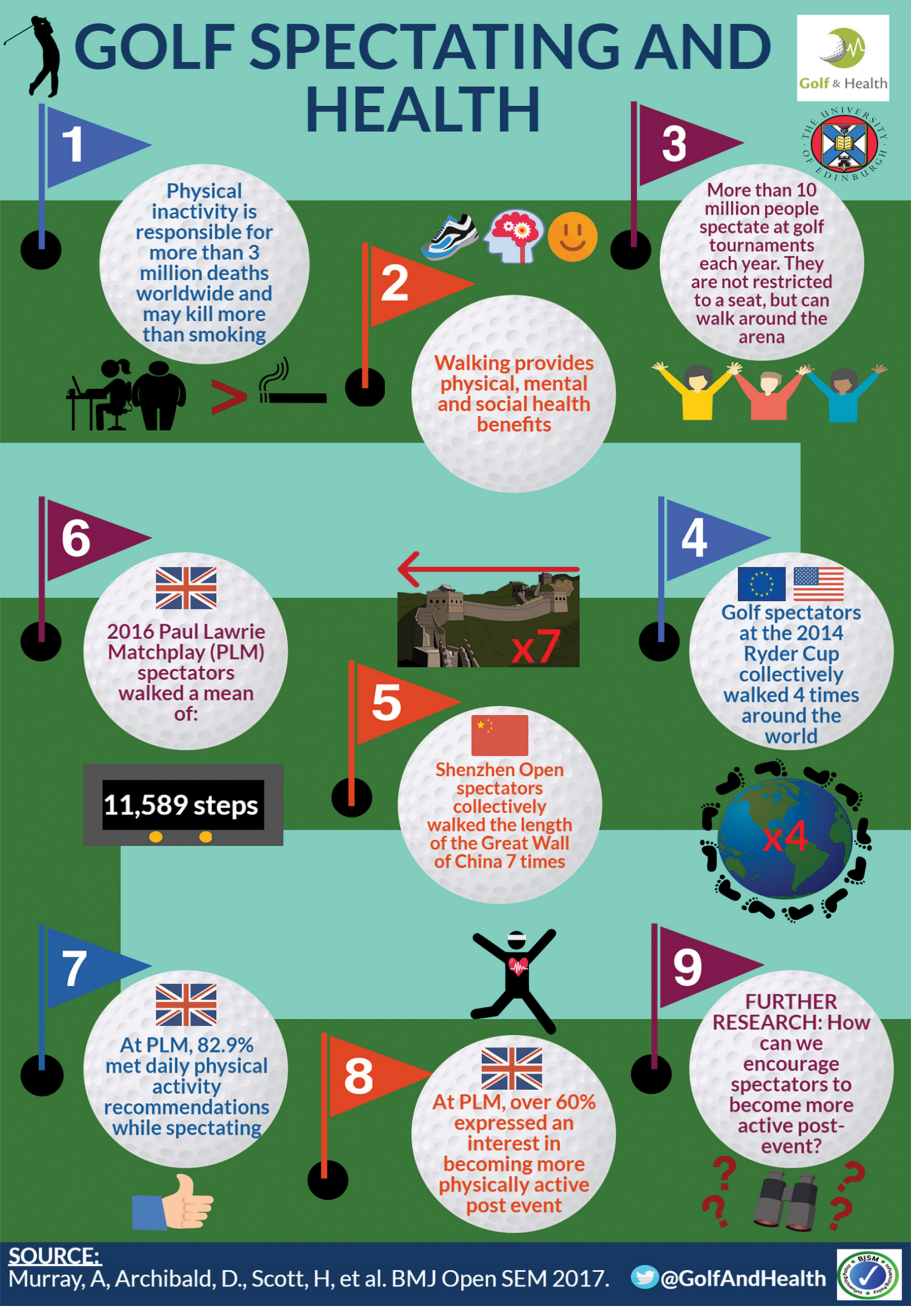# Infographic. Golf spectating and health

**DOI:** 10.1136/bjsports-2017-097933

**Published:** 2017-07-31

**Authors:** Andrew Murray, Hilary Scott, Daryll Archibald, Kieran Turner, Steffan Arthur Griffin, Chloe Schiphorst, Roger Hawkes, Paul Kelly, Liz Grant, Nanette Mutrie

**Affiliations:** 1 Physical Activity for Health Research Centre, University of Edinburgh, Edinburgh, UK; 2 Sport and Exercise, University of Edinburgh, Edinburgh, UK; 3 Faculty of Health and Social Care, Robert Gordon University, Aberdeen, UK; 4 Scottish Collaboration for Public Health Research and Policy, University of Edinburgh, Edinburgh, UK; 5 College of Medical and Dental Sciences, University of Birmingham, Birmingham, UK; 6 European Tour, European Tour Performance Institute, Viginia Water, UK; 7 Moray House School of Education, Institute of Sport Physical Education and Health Sciences, University of Edinburgh, Edinburgh, UK; 8 Global Public Health, University of Edinburgh, Edinburgh, UK

**Keywords:** Physical Activity, Golf, Exercise, Public Health, Sport

**Figure:**